# Coordinated inhibition of C/EBP by Tribbles in multiple tissues is essential for *Caenorhabditis elegans* development

**DOI:** 10.1186/s12915-016-0320-z

**Published:** 2016-12-07

**Authors:** Kyung Won Kim, Nishant Thakur, Christopher A. Piggott, Shizue Omi, Jolanta Polanowska, Yishi Jin, Nathalie Pujol

**Affiliations:** 1 Section of Neurobiology, Division of Biological Sciences, University of California San Diego, La Jolla, CA 92093 USA; 2 Centre d’Immunologie de Marseille-Luminy, Aix Marseille Université, Inserm, CNRS, Marseille, France; 3 Howard Hughes Medical Institute, University of California San Diego, La Jolla, CA 92093 USA

**Keywords:** Tribbles, C/EBP transcription factor, p38 MAP kinase, Genetic suppression, Transcriptional regulation, Signal transduction, Development, Stress responses

## Abstract

**Background:**

Tribbles proteins are conserved pseudokinases that function to control kinase signalling and transcription in diverse biological processes. Abnormal function in human Tribbles has been implicated in a number of diseases including leukaemia, metabolic syndromes and cardiovascular diseases. *Caenorhabditis elegans* Tribbles NIPI-3 was previously shown to activate host defense upon infection by promoting the conserved PMK-1/p38 mitogen-activated protein kinase (MAPK) signalling pathway. Despite the prominent role of Tribbles proteins in many species, our knowledge of their mechanism of action is fragmented, and the in vivo functional relevance of their interactions with other proteins remains largely unknown.

**Results:**

Here, by characterizing *nipi-3* null mutants, we show that *nipi-3* is essential for larval development and viability. Through analyses of genetic suppressors of *nipi-3* null mutant lethality, we show that NIPI-3 negatively controls PMK-1/p38 signalling via transcriptional repression of the C/EBP transcription factor CEBP-1. We identified CEBP-1’s transcriptional targets by ChIP-seq analyses and found them to be enriched in genes involved in development and stress responses. Unlike its cell-autonomous role in innate immunity, NIPI-3 is required in multiple tissues to control organismal development.

**Conclusions:**

Together, our data uncover an unprecedented crosstalk involving multiple tissues, in which NIPI-3 acts as a master regulator to inhibit CEBP-1 and the PMK-1/p38 MAPK pathway. In doing so, it keeps innate immunity in check and ensures proper organismal development.

**Electronic supplementary material:**

The online version of this article (doi:10.1186/s12915-016-0320-z) contains supplementary material, which is available to authorized users.

## Background

The Tribbles genes encode a family of highly conserved pseudokinases which lack key catalytic amino acids in the kinase domain [1–3]. Functional studies in multiple organisms have shown that these pseudokinases play diverse roles in innate immunity, cell signalling, energy homeostasis and cell division [2, 4]. The *Drosophila tribbles* gene is required for cell proliferation and migration in embryogenesis and oogenesis [5–8]. The mammalian Tribbles family includes three genes, *Trib1*, *Trib2* and *Trib3*, each of which plays unique roles in signalling networks regulating adipose tissue, metabolic homeostasis and the immune system [2, 4]. Abnormal function in human Tribbles has been implicated in a number of diseases including leukaemia, metabolic syndromes and cardiovascular disease [9].

Mammalian Tribbles can interact with components of the mitogen-activated protein (MAP) kinase pathway and act as adaptor proteins to modulate the strength and output of kinase signalling cascades [10–12]. Like fly Tribbles [7, 13], mammalian Tribbles bind to the basic leucine zipper (bZIP) transcription factors. Trib1 and Trib2 can induce degradation of several CCAAT/enhancer-binding protein (C/EBP) members in a context-dependent manner [1, 14, 15]. Trib3 binds to and inhibits the transcriptional activity of both activating transcription factor 4 (ATF4) and C/EBP homologous protein (CHOP) in cultured cell lines [16–19], although the in vivo functional relevance of such protein interactions remains unknown.

Given its extensive connections to different cellular processes, many questions remain to be answered regarding the role of Tribbles at the organismal level. No induction of peptide after *Drechmeria* infection 3 (NIPI-3) is the single Tribbles protein in *Caenorhabditis elegans* [20]. A role for *nipi-3* in the innate immune response was previously uncovered through the isolation of a partial loss-of-function mutation, which contains a missense mutation in the pseudokinase domain [20]*.* NIPI-3 is required for the upregulation of antimicrobial peptide (AMP) gene expression following infection by the fungus *Drechmeria coniospora* [20]. It acts upstream of a p38 MAP kinase (MAPK) pathway consisting of NSY-1/MAPKKK, SEK-1/MAPKK and PMK-1/MAPK [20, 21]. Both NIPI-3 and all components of the MAPK cascade are required cell autonomously in the epidermis during the immune response [20].

In this study, we generated null mutations of *nipi-3* and uncovered a novel role in animal development and viability. The lethality of *nipi-3* null animals is completely suppressed by loss of function in CEBP-1, a *C. elegans* member of the C/EBP family, previously known to be required for adult sensory axon regeneration and neuronal stress responses [22, 23]. Unexpectedly, loss of function in components of the PMK-1/p38 MAPK cascade also suppresses the lethality of *nipi-3* null animals. In *nipi-3* mutants, the levels of activated PMK-1 are increased, in a *cebp-1*-dependent manner. Through chromatin immunoprecipitation and deep sequencing (ChIP-seq) analyses and identification of target genes, we found that CEBP-1 binds to a conserved DNA motif. Our analyses of candidate target genes of CEBP-1 suggest a functional enrichment in development and stress responses. Importantly, in contrast to its role in innate immunity, we show that NIPI-3 acts in multiple tissues to negatively regulate transcriptional expression of *cebp-1*. This inhibition of CEBP-1 by NIPI-3 is also required across multiple tissues to enable larval development and maintain fecundity. The coordinated inhibition of CEBP-1 by NIPI-3 in multiple tissues reveals novel requirements for systemic regulation of signalling pathways in organism development.

## Results

### *C. elegans* Tribbles *nipi-3* is required for larval development and viability

**Fig. 1:**
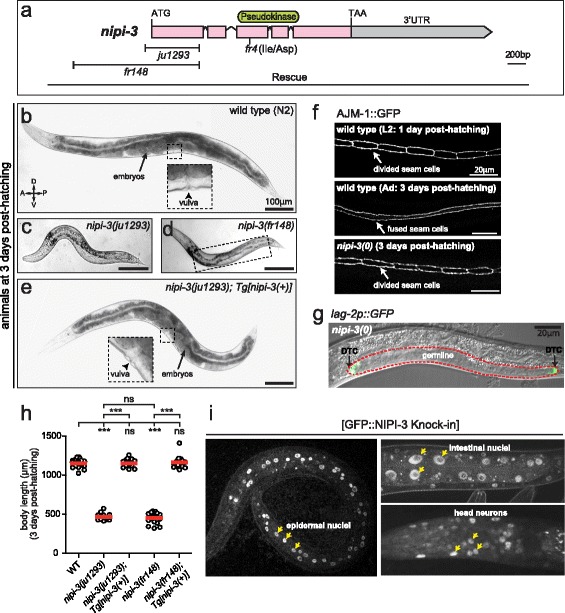
*C. elegans* Tribbles *nipi-3* is required for larval development and viability. **a** The *nipi-3* locus. *Top*, *nipi-3* encodes a pseudokinase of the Tribbles family. *Middle*, *nipi-3* deletions generated using CRISPR-Cas9 genome editing. *Bottom*, the extent of the *nipi-3* genomic region used to rescue the deletion mutants. **b**–**e** Bright-field images of worms at 3 days post-hatching; wild type (**b**), *nipi-3* null mutants (**c**,**d**) and a transgenic animal (*Tg*) expressing the wild-type *nipi-3* genomic DNA in a *nipi-3(0)* background (**e**). **f** Fluorescence images of worms expressing AJM-1::GFP reporter in the epithelial cells to allow visualization of seam cells. **g** Overlaid differential interference contrast (*DIC*) and fluorescence image of a *nipi-3(fr148)* mutant at 3 days post-hatching expressing *lag-2p::*GFP reporter. This image corresponds to the boxed region in **d**. Two distal tip cells (*DTC*) are shown in *green* (*arrow*), and the germline of arrested *nipi-3* null larva is denoted by a *dotted red line*. **h** Body length (μm) of worms at 3 days post-hatching. Each *dot* represents a single animal measured as shown; each *red line* represents the mean value. ***P < 0.001; *ns*, not significant (one-way ANOVA with Tukey’s post hoc tests). **i** Fluorescence images of endogenous NIPI-3 expression visualized in the GFP KI strain (*fr152)*. Expression is observed in the nuclei (*yellow arrows*) of the epidermis (*left panel*), the intestine (*upper right panel*) and the head neurons (*lower right panel*)

To better understand the biological roles of *nipi-3*, we used clustered regularly interspaced short palindromic repeats (CRISPR)-Cas9 genome editing [24–27] to generate two deletion alleles of *nipi-3* (*fr148* and *ju1293*) and a green fluorescent protein (GFP) knock-in (KI) (*fr152)* (Fig. 1a; see Methods and below). The *fr148* and *ju1293* deletion alleles remove 1.6 kb and 0.6 kb of the 5′ region of the gene, respectively (Fig. 1a), resulting in molecular nulls for *nipi-3*. The phenotypes of homozygous mutants of either *nipi-3* deletion allele, designated as null (0), were indistinguishable (Fig. 1c, d). Mutants arrested development at the second to third larval stages (L2–L3) (see below) and eventually died between 5–10 days after hatching. When compared to wild-type larvae at the same stage (2 days post-hatching), *nipi-3(0)* arrested larvae displayed a small and dumpy body morphology. At 3 days post-hatching, wild-type animals reached the adult stage, as evidenced by fusion of seam cells (lateral epidermal cells), formation of adult alae and the vulva (Fig. 1b, f). By contrast, all age-matched *nipi-3(0)* animals were arrested at L2–L3, as the seam cells did not fuse, adult alae were not observed and the vulval invagination did not occur (Fig. 1c, d, f). In these mutant animals, the germline also appeared to be arrested, generally at L3 based on the size of the gonad and the number of germ cells (Fig. 1c, d, g). Occasionally, in *nipi-3(0)* animals with longer bodies, we observed some sperm or a few unfertilized oocytes. The *nipi-3(0)* animals also exhibited an abnormal pharyngeal morphology (Additional file 1: Figure S1). We rescued the larval lethality and sterility of *nipi-3(0)* by expressing the wild-type *nipi-3* genomic DNA as high-copy-number extrachromosomal arrays (Fig. 1a, e, h; Methods). As expression from such transgenes is silenced in the germline [28], this result indicates that the larval lethality and germline development defects of *nipi-3(0)* are both primarily due to its function in somatic tissues. Thus, the analyses of *nipi-3* null animals indicate an essential somatic role of *nipi-3* in organism development.

Knocking-in *gfp* to the *nipi-3* locus, which produced a protein tagged at its N-terminus (GFP::NIPI-3), had no adverse effect; KI animals (*fr152)* were fully viable and indistinguishable from wild type in growth and movement. We observed GFP expression in the epidermis, intestine and in neurons (Fig. 1i), consistent with the previously reported expression pattern obtained using transgenic transcriptional reporters [20]. Interestingly, although NIPI-3 does not have a clearly identifiable nuclear-localization signal, GFP::NIPI-3 expression was observed predominantly in the nuclei (Fig. 1i), with an overall intensity peaking at the L2–L3 stages. This nuclear localization suggests a role for NIPI-3 in regulation of gene expression.

### Loss of *cebp-1* suppresses the lethality, but not the innate immune response defect, of *nipi-3* null animals

To dissect the molecular mechanisms involving NIPI-3, we undertook a yeast two-hybrid screen using the full-length NIPI-3 protein as bait (Cypowyj S. et al., manuscript in preparation). One prominent candidate interacting partner was CEBP-1, a member of the C/EBP family of transcription factors [22]. The CEBP-1 protein consists of 319 amino acids, with a bZIP domain at its C-terminus. In further analyses using the yeast two-hybrid assay, we found that the N-terminal region (amino acids 1–115) of CEBP-1 was sufficient for binding NIPI-3 (Additional file 2: Figure S2). This interaction is reminiscent of those observed for fly and vertebrate Tribbles proteins, which bind and degrade C/EBP family proteins [1, 7, 13–15, 29], with human Trib1 binding the N-terminus of C/EBPα [1]. Thus, the ability of Tribbles and C/EBP proteins to interact directly is likely conserved from *C. elegans* to humans.

**Fig. 2:**
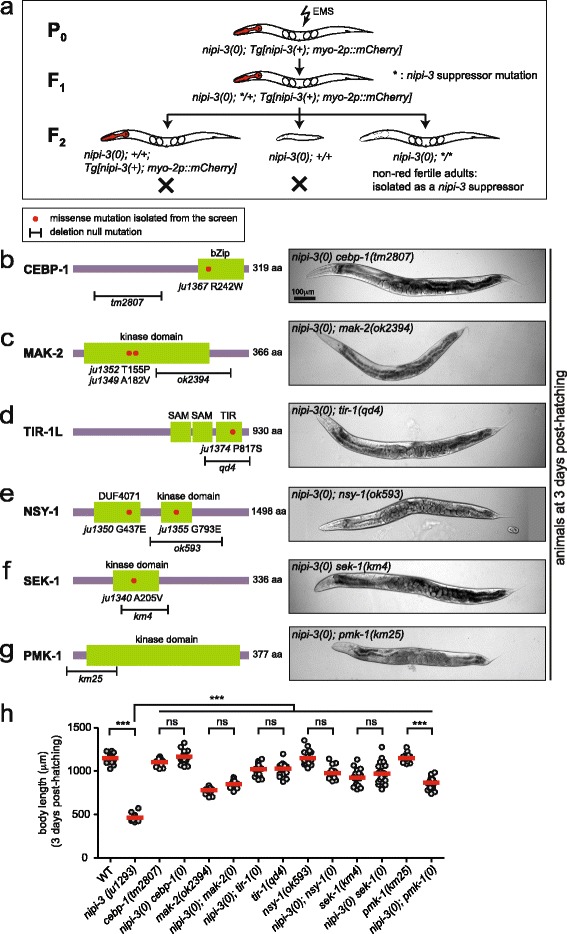
*nipi-3(0)* lethality is suppressed by loss of *cebp-1* or components of a PMK-1/p38 MAPK cascade. **a** Schematic overview of the forward genetic screen designed to identify the suppressors of *nipi-3(0)* larval arrest and lethality. **b**–**g** The mutations isolated from the *nipi-3(0)* suppressor screen and the deletion null mutations tested for *nipi-3(0)* suppression assay are shown in the *left column* and bright-field images of worms at 3 days post-hatching are shown in the *right column*. **h** Body length (μm) of worms at 3 days post-hatching. Each *dot* represents a single animal measured as shown; each *red line* represents the mean value; some data are replicated from Fig. 1 as shown with *darker grey* dots. ***P < 0.001; *ns*, not significant (one-way ANOVA with Tukey’s post hoc tests)

To understand the functional significance of the observed protein interaction, we next performed genetic analysis using null mutations of *nipi-3* and *cebp-1.* Null mutants of *cebp-1* show normal development and body appearance. Remarkably, *cebp-1(0)* completely suppressed the growth and fertility defects of *nipi-3(0)* mutants (Fig. 2b, h; Methods). *cebp-1(0)* also completely suppressed the body size defect and the developmental delay observed at 25 °C of the partial loss-of-function *nipi-3(fr4)* mutants (Additional file 3: Figure S3). NIPI-3 is also known to be necessary for the epidermal innate immune response upon fungal infection [20]. The expression of AMP genes after fungal infection is highly induced in wild-type animals and abrogated in *nipi-3(fr4)* mutants [20]. However, *cebp-1(0) nipi-3(0)* animals did not exhibit an induction of the AMP gene *nlp-34* upon infection (Additional file 4: Figure S4a). Further, *cebp-1(0) nipi-3(0)* animals expressing wild-type *cebp-1* in a tissue-specific manner in either the epidermis or neurons also showed no AMP gene induction (Additional file 4: Figure S4a). These results indicate that loss of *cebp-1* does not suppress the immune response defect in *nipi-3(0)*. As *cebp-1(0)* strongly impairs sensory axon regeneration after laser axotomy [22], we also tested whether *nipi-3* affected posterior lateral microtubule (PLM) axon regeneration. Neither *nipi-3(0)* nor *nipi-3(fr4)* showed significant effects in axon regeneration, and *cebp-1(0) nipi-3(0)* double mutants showed impaired axon regeneration similar to *cebp-1(0)* (Additional file 4: Figure S4b). These results show that the genetic interaction between *nipi-3* and *cebp-1* is highly specific for larval development and organism fecundity.

### Loss of the PMK-1/p38 MAPK pathway also suppresses *nipi-3(0)* lethality

To gain further insight into the mechanism underlying *nipi-3*’s role in animal development, we performed a forward genetic screen for suppressors of *nipi-3(0)* lethality. We mutagenized *nipi-3(ju1293); Tg[nipi-3(+); myo-2p::mCherry]* animals (Methods). Among their F_2_ progeny, we isolated fertile animals that had lost the rescuing transgene (Fig. 2a) and established multiple suppressor lines (genotypes designated as *nipi-3(0); suppressor).* We screened ~16,000 haploid genomes and identified 7 independent suppressor alleles. All the identified suppressors in this study were associated with a full reversion of the *nipi-3(0)* lethality; fertile adults could be propagated without the *nipi-3(+)* rescuing transgene.

We characterized several suppressor mutations using candidate gene analyses in combination with whole genome sequencing. One suppressor caused a missense mutation in the bZIP domain of *cebp-1,* and behaved like *cebp-1(0)* (Fig. 2b, h). Among the other suppressors of *nipi-3(0)*, we found missense alterations in TIR-1/the sterile alpha (SAM) and Toll-interleukin receptor (TIR) motif-containing protein SARM), NSY-1/MAPKKK, SEK-1/MAPKK and MAK-2/MAPK-activated protein kinase (MAPKAPK) (Fig. 2c–f). These mutations are located in known functional domains, including the TIR domain for TIR-1*,* the kinase domains for NSY-1, SEK-1 and MAK-2, and the DUF4071 domain, commonly found at the N-terminus of many serine-threonine kinase-like proteins, for NSY-1 (Fig. 2c–f). We then tested known null mutants in each of these genes and found them to suppress *nipi-3(0)* to the same degree as our suppressor mutations, as shown by quantification of the body length (Fig. 2b–f, h; Additional file 5: Figure S5a). Thus, loss of function in *tir-1* and these three kinase genes causes strong suppression of *nipi-3(0).*

NIPI-3 has a specific role in the regulation of epidermal defense genes. It acts together with TIR-1, NSY-1 and SEK-1, as well as several other genes including *tpa-1*, *pmk-1* and *sta-2* [20, 21, 30]. We therefore tested mutants for these three latter genes for their genetic interaction with *nipi-3(0).* We found that loss of *pmk-1,* but not *tpa-1* or *sta-2,* suppressed the lethality of *nipi-3(0)* (Fig. 2 g, h for *pmk-1*; Additional file 5: Figure S5b for *tpa-1* and *sta-2*)*.* Loss of *pmk-1* resulted in suppression of *nipi-3(0)* phenotypes similar to the other suppressor mutants such that *nipi-3(0); pmk-1(0)* double mutants developed into fertile adults with adult alae and vulva. The rescue of body size, however, was not complete (Fig. 2 g, h). As *cebp-1* is involved in two other MAPK cascades known for their roles in adult axon regeneration [22, 31, 32], we tested mutations in several other candidate genes and found that loss of function in *dlk-1, pmk-3, mlk-1* or *kgb-1* did not suppress *nipi-3(0)* defects (Additional file 5: Figure S5b). Together, our analyses from both the forward genetic screening and test of candidate mutants reveal a previously unknown role of CEBP-1 in larval development mediated by NIPI-3, and a novel genetic interaction between NIPI-3 and the PMK-1/p38 MAPK cascade.

### NIPI-3 inhibits PMK-1 phosphorylation via CEBP-1

**Fig. 3:**
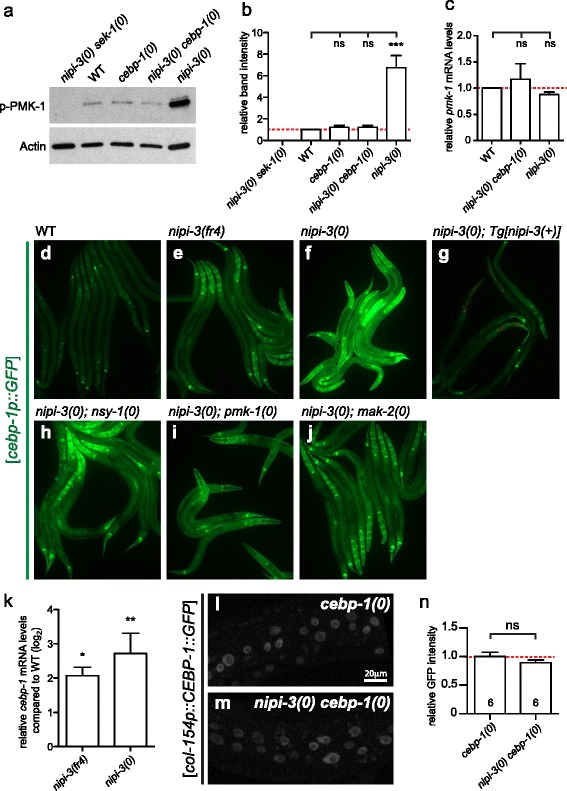
NIPI-3 represses PMK-1 phosphorylation via *cebp-1* and represses *cebp-1* transcription. **a** Western blot analysis on total protein lysate from various animal strains using the indicated antibodies, α-phospho-p38 MAPK antibody to detect a phosphorylated form of PMK-1 proteins (*p-PMK-1*) or α-actin antibody as a loading control. **b** Densitometric quantifications of immunoblot signals normalized to actin. *n* = 3; error bars represent standard error of the mean (*SEM*); *** P < 0.001 (one-way ANOVA with Tukey’s post hoc tests). **c** Quantitative RT-PCR (qRT-PCR) analysis of *pmk-1*. Relative abundance of *pmk-1* mRNA normalized to *actin* mRNA. *n =* 3; error bars represent SEM; *ns*, not significant (one-way ANOVA with Tukey’s post hoc tests). **d**–**j** Fluorescence images of *cebp-1p::GFP* reporter animals at L2. **k** qRT-PCR analysis of *cebp-1* in WT, *nipi-3(fr4), nipi-3(0)* animals. Relative abundance of *cebp-1* mRNA normalized to *actin* mRNA. *n =* 3; error bars represent SEM; *P < 0.05; **P < 0.01; *ns*, not significant (one-way ANOVA with Tukey’s post hoc tests). **l**, **m** Confocal fluorescence images (z-stack) of *col-154p(epidermis)::CEBP-1::GFP* reporter animals at L2. **n** Quantification of GFP intensity measured in each epidermal nucleus (10 per animal). *n* = 6; error bars represent SEM; *ns*, not significant (Student’s unpaired *t* test). Primary data for panels (**b**, **c**, and **k**) are provided in Additional file 14

To dissect the mechanism underlying the interaction between PMK-1/p38 MAPK and NIPI-3, we first asked how the levels of active PMK-1 might be altered in *nipi-3(0)* suppressor animals. We performed a Western blot analysis using an anti-phospho-p38 MAPK antibody that specifically recognizes phosphorylated, active PMK-1 [30]. We made protein lysates from animals at 1 day post-hatching (L2) because *nipi-3(0)* animals at this stage were as healthy as the wild type. We observed that levels of active PMK-1 were significantly increased in *nipi-3(0)*, but remained similar to wild type in *cebp-1(0)* and *cebp-1(0) nipi-3(0)* animals (Fig. 3a, b). Phosphorylated PMK-1 was undetectable in *nipi-3(0) sek-1(0)* animals, consistent with PMK-1 being activated by SEK-1 (Fig. 3a, b). The total PMK-1 levels were likely unchanged in *nipi-3(0)*, as the mRNA levels of *pmk-1* were comparable between *nipi-3(0)* and wild type when assessed by quantitative RT-PCR (Fig. 3c)*.* We note that *mak-2(0)* did not affect phosphorylated PMK-1 (Additional file 6: Figure S6), suggesting that MAK-2 likely acts downstream of, or in parallel to, PMK-1. Together, these results suggest that the abnormally high levels of phosphorylated PMK-1 in *nipi-3(0)* are dependent on *cebp-1*.

### NIPI-3 represses the transcription of *cebp-1* in multiple tissues

To dissect how NIPI-3 inhibits CEBP-1, we examined whether *cebp-1* levels were altered in *nipi-3(0)* and in each *nipi-3(0)* suppressor. Transcriptional reporters of *cebp-1* (*Tg[cebp-1p::GFP]*) were broadly expressed in most post-embryonic tissues, including epidermis, muscles, pharynx, intestine and neurons (Fig. 3d–j). Strikingly, the expression of the *cebp-1* transcriptional reporter was highly and significantly increased in both *nipi-3(fr4)* and *nipi-3(0)* mutants, compared with wild-type animals (Fig. 3d–f). Quantitative RT-PCR analysis also showed significantly increased expression of *cebp-1* mRNAs in *nipi-3* mutant animals (Fig. 3 k). Consistent with the observed transcriptional regulation, we found that a translational CEBP-1::GFP reporter driven by a heterologous epidermal promoter showed no detectable differences in GFP expression in a *nipi-3(0)* background (Fig. 3 l–n). The increased expression of *cebp-1p::GFP* in *nipi-3(0)* was reduced to normal levels when a *nipi-3(+)* transgene was introduced (Fig. 3 g), indicating that NIPI-3 represses the transcription of *cebp-1*. Additionally, the transcriptional repression of *cebp-1* by NIPI-3 was largely independent of the PMK-1 pathway since *cebp-1p::GFP* expression in *nipi-3(0)* remained high in animals that also carried a null mutation of *nsy-1, pmk-1* or *mak-2* (Fig. 3 h–j). Together, the results show that NIPI-3 negatively regulates expression of *cebp-1* at the transcriptional level and that CEBP-1 acts upstream of the PMK-1 pathway.

The tight regulation of *cebp-1*’s expression level is critical for animal viability. We found that suppression of *nipi-3(0)* by *cebp-1(0)* was semi-dominant, as *cebp-1(0)/+* caused partial but significant suppression of the short body length of *nipi-3(0)* mutants (Additional file 7: Figure S7). Moreover, in a wild-type background, the transgene *eft-3p::CEBP-1::GFP,* which drives strong and ubiquitous expression of CEBP-1, caused dose-dependent lethality (see Methods). In addition, expression of a full-length functional translational reporter of *cebp-1* (*cebp-1p::CEBP-1::GFP*) in *nipi-3(0)* mutant animals exacerbated developmental defects and accelerated larval lethality, while the same transgene showed no such effects in a wild-type background. Together, these results support the conclusion that the lethality observed in *nipi-3(0)* mutants is a direct consequence of *cebp-1* overexpression.

### Overexpression of truncated forms of CEBP-1 suppresses *nipi-3(0)* lethality

**Fig. 4:**
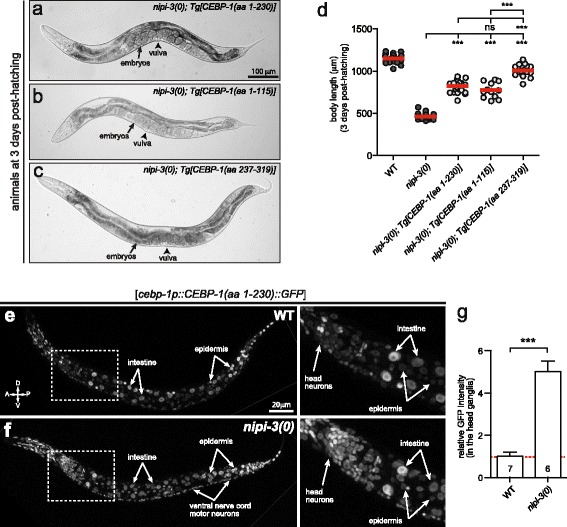
Overexpression of truncated forms of CEBP-1 protein suppresses *nipi-3(0)* lethality. **a**–**c** Bright-field images of worms at 3 days post-hatching expressing truncated forms of CEBP-1 proteins. **d** Body length (μm) of worms at 3 days post-hatching. Each *dot* represents a single animal measured as shown; each *red line* represents the mean value; some data are replicated from Fig. 1 as shown with *darker grey dots*. ***P < 0.001; *ns*, not significant (one-way ANOVA with Tukey’s post hoc tests). **e**, **f** Confocal fluorescence images (z-stack) of *cebp-1p::CEBP-1(aa 1-230)::GFP* reporter animals at L2. **g** Quantification of GFP intensity measured in the region of head neurons. Error bars represent SEM; ***P < 0.001 (Student’s unpaired *t* test)

To dissect the molecular basis of CEBP-1’s role in animal development, we expressed truncated forms of CEBP-1 lacking the bZIP domain (amino acids 1–230 or 1–115), or lacking the N-terminus (amino acids 237–319) in *nipi-3(0)* mutants. Surprisingly, in stark contrast to the strong lethality caused by overexpressing full-length CEBP-1 in *nipi-3(0)* mutants, we found that expression of either the N-terminal fragment or the bZIP domain of CEBP-1 alone resulted in significant suppression of *nipi-3(0)* defects (Fig. 4a–d). Overexpression of either the N- or C-terminal truncated protein caused no defects either in the wild-type or *cebp-1(0)* backgrounds. Among the three CEBP-1 fragments, the expression of C-terminal CEBP-1 (amino acids 237–319) showed the most effective rescue, judged by the fecundity of the transgenic lines and quantitative comparisons of body length (Fig. 4c, d). The expression levels of CEBP-1(amino acids 1–230)::GFP were markedly increased in *nipi-3(0)*, presumably reflecting the transcriptional regulation of *cebp-1* described above. We noticed the fluorescence intensity was most strongly increased in the epidermis and neurons, throughout the head region and in the ventral nerve cords (Fig. 4e–g). These observations suggest that the truncated forms of CEBP-1 act in a dominant negative manner to inhibit the activity of the endogenous CEBP-1.

### CEBP-1 binds conserved DNA motifs in genes regulating development and stress response

To gain further insight into CEBP-1’s function in animal development, we next sought candidate target genes of CEBP-1 by performing ChIP-seq analysis on transgenic animals expressing a functional FLAG-tagged CEBP-1 protein in a *cebp-1(0)* background (Methods). We found 209 CEBP-1 ChIP-seq peaks in the genome that were associated with 212 coding genes (Additional file 8: Table S1). CEBP-1 peaks were preferentially located within the promoter regions of the target genes (169 genes, 79 %), less frequently within introns (43 genes, 21 %) and never within exons.

**Fig. 5:**
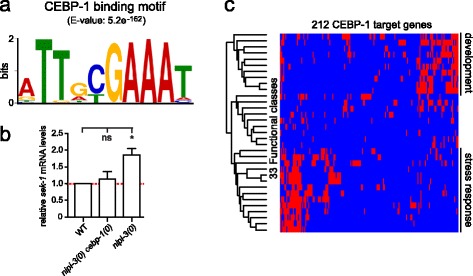
CEBP-1 binds conserved DNA motifs in genes regulating development and stress response. **a** Motif logo of the most over-represented motif among CEBP-1 ChIP-seq peaks. **b** qRT-PCR analysis of *sek-1*. Relative abundance of *pmk-1* mRNA normalized to *actin* mRNA. *n =* 3; error bars represent SEM; *P < 0.05; *ns*, not significant (one-way ANOVA with Tukey’s post hoc tests). Primary data are provided in Additional file 14. **c** Hierarchical clustering of genes and functional classes (see Additional file 10: Figure S9 and Additional file 8: Table S1 for class labels and full data); the presence of a gene in a class is represented by a *red rectangle*, its absence by *blue*

We then performed motif analysis of the genomic regions bound by CEBP-1 using the motif discovery tools Multiple Em for Motif Elicitation (MEME) [33] and Regulatory Sequence Analysis Tools (RSAT) [34]. The most over-represented motif, NTTDYGAAAH, was found in 139 out of 209 CEBP-1 ChIP-seq peak regions (Fig. 5a). We then compared this motif with published motifs using the motif comparison tool Tomtom [35] and found the most statistically significant similarities to vertebrate C/EBP binding motifs [36]. The conservation of CEBP-1 binding motif further reinforces the functional parallels between *C. elegans* CEBP-1 and vertebrate C/EBPs.

As CEBP-1 likely acts upstream of the PMK-1 pathway, we searched among the targets of CEBP-1 for components of the PMK-1 pathway and found CEBP-1 ChIP-seq peaks present in the promoter of *sek-1* (Additional file 9: Figure S8). When we examined the mRNA levels of *sek-1* by quantitative RT-PCR in *nipi-3(0)* animals where *cebp-1* is overexpressed, we observed increased *sek-1* mRNA levels (Fig. 5b). In contrast, in *nipi-3(0) cebp-1(0)* animals the levels of *sek-1* mRNAs were similar to those of the wild type. These results suggest that the abnormally high levels of *cebp-1* in *nipi-3(0)* can promote the expression of *sek-1*, which in turn promotes the phosphorylation of PMK-1 [30], leading to abnormal larval development and lethality.

We then asked whether the list of potential CEBP-1 targets was enriched for genes with specific functions. To this end, we searched for enriched categories through an Expression Analysis Systematic Explorer (EASE) analysis [37, 38], using our in-house database of functional annotations as described [39]. This includes 4600 datasets automatically updated from multiple sources including WormBase, FlyBase, the Kyoto Encyclopedia of Genes and Genomes (KEGG) and relevant RNAi databases. Most (80 %) of CEBP-1 target genes were associated with at least one of 33 enriched functional classes (*P* < 10^-5^; Additional file 8: Table S1). Hierarchical clustering of the genes in each of the enriched classes identified two main groups (Fig. 5c; Additional file 10: Figure S9; Additional file 8: Table S1). One group is related to development with phenotypic classes such as 'larval lethal’ or 'slow growth’ and includes genes involved in basic cellular processes, transcription, translation or endocytosis. The second group is related to the response to biotic or abiotic stress, including response to cadmium, hygromycin or bacterial toxins. Interestingly, McEwan et al. have found that most of the genes upregulated in *nipi-3(fr4)* mutants are also induced by the translational inhibitory toxin ToxA. Out of the 14 stress-related classes associated with the *cebp-1* targets, 10 are shared with those found for genes upregulated in *nipi-3(fr4)* (D.L. McEwan, personal communication; *P* < 10^-10^; Additional file 8: Table S1). On the other hand, consistent with the fact that the *nipi-3(fr4)* allele does not provoke larval lethality, only 1 out of the 10 classes in the development cluster is shared between the *cebp-1* targets and the genes upregulated in *nipi-3(fr4)*. These analyses suggest that CEBP-1 regulates genes functioning in development and in stress responses, and might have a particularly important impact on organismal physiology when overexpressed in a *nipi-3* mutant context.

### NIPI-3 is required in multiple tissues to ensure proper larval development

**Fig. 6:**
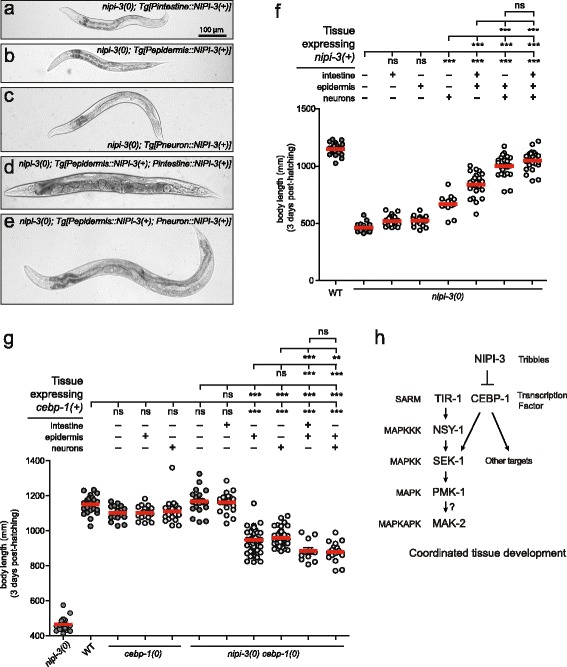
Tight regulation of both NIPI-3 and CEBP-1 is required in multiple tissues for proper organism development. **a**–**e** Bright-field images and (**f**) the body length of worms expressing tissue-specific *nipi-3(+)* driven by the intestinal (*mtl-2*), epidermal (*col-12*) or pan-neuronal (*rgef-1*) promoters in a *nipi-3(0)* background. **g** The body length of worms expressing tissue-specific *cebp-1(+)* driven by the intestinal (*ges-1*), epidermal (*col-154*) or pan-neuronal (*rgef-1*) promoters in a *nipi-3(0) cebp-1(0)* background. **f**, **g** Each *dot* represents a single animal measured as shown; each *red line* represents the mean value; some data are replicated from Figs. 1 and 2 as shown with *darker grey dots*. **P < 0.01; ***P < 0.001; *ns*, not significant (one-way ANOVA with Tukey’s post hoc tests). **h** Working model for NIPI-3 function in *C. elegans* development. In wild type, presence of NIPI-3 keeps *cebp-1* expression level optimal for coordinated tissue development. In *nipi-3(0),* however, *cebp-1* and *sek-1* are overexpressed and in turn PMK-1 is hyperactivated

We next asked in which tissue the expression of *nipi-3(+)* is required for animal viability. We expressed *nipi-3(+)* in a tissue-specific manner, using intestinal, epidermal and pan-neuronal promoters, in the *nipi-3(0)* background (Fig. 6a–f). In contrast to the complete rescue of body size and lethality in *nipi-3(0)* mutants expressing *nipi-3(+)* under its own promoter (Fig. 1e, h), expressing *nipi-3(+)* in individual tissues failed to rescue the developmental arrest (Fig. 6a–c, f). Pan-neuronal expression of *nipi-3(+)* resulted in slightly increased body length of *nipi-3(0)* 3 days post-hatching (Fig. 6c, f). Since *nipi-3* activity strongly inhibits *cebp-1* transcription in the epidermis and neurons (Fig. 4e–g), we expressed *nipi-3(+)* in both of these tissues together, and found that these transgenic animals showed an increased body size (Fig. 6e, f), compared to those expressing *nipi-3(+)* in each tissue alone, or in the intestine and epidermis simultaneously (Fig. 6a–d, f). Some of the transgenic animals with a body length closer to that of wild-type animals showed improved somatic and germline development, with formation of vulva and adult alae, and produced a few viable but infertile progeny. When we expressed *nipi-3(+)* in all three tissues together, the transgenic animals showed no further improvement of body size compared to those expressing *nipi-3(+)* in both the epidermis and neurons (Fig. 6f)*,* and did not recapitulate the rescue of lethality associated with the expression of *nipi-3* under its own promoter. Thus, we conclude that NIPI-3 is required in multiple tissues, particularly epidermis and neurons, for animal growth and development.

### Suppression of *nipi-3(0)* by *cebp-1(0)* also requires a block of CEBP-1 activity in multiple tissues

Conversely, we asked whether expression of *cebp-1(+)* in a single tissue might cause the viable *nipi-3(0) cebp-1(0)* animals to die. We expressed *cebp-1(+)* using the intestinal, epidermal and pan-neuronal promoters in *cebp-1(0) nipi-3(0)* double mutants (Fig. 6 g). Expression of *cebp-1(+)* in individual tissues was insufficient to produce larval lethal phenotypes in *cebp-1(0) nipi-3(0)* animals. Interestingly, epidermal or neuronal expression of *cebp-1(+)* in *cebp-1(0) nipi-3(0)* double mutants caused short body length, but not in *cebp-1(0)* mutants (Fig. 6 g). Co-expression of *cebp-1(+)* in the epidermis and neurons resulted in further reductions in body length, although these were not as severe as those in *nipi-3(0)* animals (Fig. 6 g). In addition, we noticed that the same transgenes expressing *cebp-1(+)* in both epidermis and neurons caused an abnormal pharyngeal morphology in *cebp-1(0) nipi-3(0)* animals, similar to that seen in *nipi-3(0)* mutants (Additional file 1: Figure S1). Together, our data suggest that the tight regulation of both NIPI-3 and CEBP-1 in multiple tissues is required in a systemic manner for normal animal growth and development.

## Discussion

*C. elegans* Tribbles NIPI-3 was identified on the basis of its roles in host defense [20, 21]. Here, through generation and analyses of null alleles, we find *nipi-3* to be essential for animal development and viability. Remarkably, the larval arrest and lethality caused by complete loss of *nipi-3* is fully suppressed by loss of *cebp-1*, a C/EBP bZIP transcription factor, or by loss of function in the PMK-1/p38 MAPK cascade including *tir-1/*SARM, *nsy-1/*MAPKKK, *sek-1*/MAPKK and *pmk-1*/MAPK*.* Our data show that complete elimination of the function of *nipi-3* causes abnormally high expression of CEBP-1, and activation of PMK-1 MAPK. This then disrupts development and leads to death. The level of *sek-1* mRNA is increased in *nipi-3(0)* mutants but not in *cebp-1(0)* or in *nipi-3 cebp-1* animals. The level of phosphorylated (active) PMK-1 follows the same trend. Coupled with our ChIP-seq analyses and genetic epistasis data, this suggests that CEBP-1 acts as a direct positive regulator of *sek-1*. The PMK-1 pathway is therefore activated when CEBP-1 expression is high in *nipi-3(0).* On the other hand, *cebp-1* expression levels remain high in *nipi-3(0); pmk-1(0)* animals, confirming that CEBP-1 does not act downstream of the PMK-1 pathway. Together, these results suggest that NIPI-3 negatively regulates the PMK-1 MAPK cascade, via CEBP-1, to promote animal viability and development (Fig. 6 h).

In innate immunity, however, *nipi-3* cell-autonomously promotes or enhances the same p38 kinase cascade to activate host defense in the epidermis [20]*.* It has been shown that overexpression of *sek-1* in the epidermis rescues the block of AMP induction in *nipi-3* mutants upon fungal infection [20], and an overexpression of *nipi-3* provokes an increase in the constitutive expression of AMP which is dependent on the p38 cascade [21]. It is intriguing that NIPI-3 appears to be capable of activating or inhibiting PMK-1/p38 in the epidermis at different times or under different conditions (infection versus development). How might NIPI-3 achieve this dual role under different stresses and in altered cellular contexts? As Tribbles proteins are well known to act as adaptors, NIPI-3 might be regulated via binding with other co-factors only present under specific circumstances. Indeed, we find that other upstream and downstream components of the epidermal immune response cascade are not involved in the developmental regulation described here. Thus, the core PMK-1/p38 MAPK cassette has evolved context-specific functions depending on different upstream regulators or co-factors [40, 41]. Members of the Tribbles family in other species have been mostly studied in the context of cell proliferation, adipocyte tissue differentiation, energy metabolism and immunity, where they function in a cell-autonomous manner. Our discovery of the opposing roles of NIPI-3 in development and in the immune response illustrates how cellular context can alter the function of highly conserved signalling molecules.

Negative regulation of C/EBP by Tribbles has been observed throughout the animal kingdom. *Drosophila* and mammalian Tribbles bind and degrade C/EBP proteins [1, 7, 13–15]. We find that *C. elegans* NIPI-3 represses the transcription of *cebp-1*, which has important functional consequences in vivo. This form of regulation has not been reported in other organisms. Given its nuclear localization, NIPI-3 may inhibit the transcription of *cebp-1* by interfering with other transcription factor(s). The promoter of *cebp-1* contains putative CEBP-1 binding consensus motifs, raising the possibility that NIPI-3, by binding to CEBP-1, may also alter the transcriptional activity of CEBP-1.

NIPI-3 is required to control CEBP-1 levels in multiple tissues for animal development and viability. Consistent with the inhibition of CEBP-1 expression by NIPI-3 in the epidermis and neurons, simultaneous expression of *nipi-3(+)* in both tissues makes a noticeable contribution to animal development in *nipi-3(0)* mutants, compared with *nipi-3(+)* expression in single tissues. Conversely, simultaneous expression of *cebp-1(+)* in both epidermis and neurons causes noticeable defects in animal development in *nipi-3(0) cebp-1(0)* mutants, compared with *cebp-1(+)* expression in single tissues. Thus, a tightly regulated coordination of these two genes’ interactions in multiple tissues is required to ensure proper development.

A key conclusion from our study is that the precise control of CEBP-1 and PMK-1/p38 MAPK pathways in multiple tissues is critical for organismal development. NIPI-3 acts as a master regulator to prevent improper activation of CEBP-1 and PMK-1, whose hyperactivation during development has deleterious consequences. Interestingly, hyperactivation of PMK-1/p38 was previously shown to block larval development when the endoplasmic reticulum unfolded protein response was altered [42]. Moreover, innate immune activation with a xenobiotic that provides protection from bacterial infection in the adult has been shown to provoke a growth delay during development [43]. Subsequently, an elegant genetic suppressor screen revealed that mutations in the PMK-1/p38 MAPK pathway suppressed this developmental phenotype [44]. Thus, the NIPI-3/CEBP-1 axis is a key mechanism by which immune effector expression is held in check during nematode development.

During normal development, both CEBP-1 and PMK-1 are maintained at a basal level by NIPI-3. The levels of inducible signalling from these pathways are, however, important for animals to protect themselves or to promote repair. For instance, following fungal infection, NIPI-3 promotes the PMK-1/p38 MAPK signalling pathway in the epidermis [20]. Thus, animals can successfully defend themselves from fungal infection with activated PMK-1 locally in the epidermis, while survival is not affected as PMK-1 remains inactive in other tissues. Similarly, CEBP-1 is known to play a key role in neuronal stress responses [22, 23, 45], and we identified potential CEBP-1 target genes that are involved in different stress responses. Moreover, a concomitant study has identified NIPI-3 as a negative regulator of CEBP-1 in intestinal defense against the bacterial toxin ToxA (McEwan et al., personal communication). An important challenge for the future will be to understand how NIPI-3 regulates its downstream pathways and how NIPI-3 itself is regulated depending on developmental and environmental conditions. Understanding the molecular mechanism of this systemic, coordinated regulation should advance our knowledge of how animal development can be maintained in the face of environmental stresses.

## Conclusions

We showed a novel essential role for the *C. elegans* Tribbles homolog NIPI-3 in animal development and viability, which requires NIPI-3’s function in multiple tissues. NIPI-3 acts as a master regulator to prevent improper activation of a C/EBP transcription factor and a conserved PMK-1/p38 MAPK signalling cascade known to control innate immunity. These findings suggest that innate immune responses are tightly controlled for proper organismal development.

## Methods

### Strains, transgenes and plasmids

*C. elegans* strains were maintained under standard conditions at 20 °C unless otherwise mentioned. The wild type was the N2 Bristol strain [46]. New strains were constructed using standard procedures, and all genotypes were confirmed by PCR or sequencing. All strains and their genotypes used in this study are described in Additional file 11: Table S2. Extrachromosomal array transgenic lines were generated as described previously [47]. Expression constructs, transgenes and strain genotypes are also summarized in Additional file 11: Table S2. For all experiments, at least two independent transgenic lines were examined and quantitative data are shown for one. In our studies of *cebp-1* dosage effect, we could not generate transgenic animals when injecting 10 ng/μl of *eft-3p::CEBP-1::GFP* transgenes into wild-type animals, while many transgenic lines were obtained when *cebp-1::gfp* DNA was injected with lower concentrations (i.e. 1 ng/μl or 0.01 ng/μl). All plasmids used in this study are described in Additional file 12: Table S3a.

### CRISPR-Cas9-mediated deletion and GFP KI

We generated the *nipi-3(ju1293)* deletion allele using the co-CRISPR method [25, 48]. We used four single guide RNAs (sgRNAs) targeting the N-terminus of the *nipi-3* gene (Additional file 12: Table S3a). *U6p::nipi-3* sgRNAs were generated by Gibson assembly and injected into *cebp-1(tm2807)* worms, using standard methods, in mixtures composed of 30 ng/μl of each *nipi-3* sgRNA, 50 ng/μl of *eft-3p::Cas9-SV40NLS::tbb-2 3′UTR,* 50 ng/μl of *U6p::unc-22* sgRNA and 1.5 ng/μl of *myo-2p::mCherry.* For the *nipi-3(fr148)* deletion allele, a single sgRNA targeting the N-terminus of the *nipi-3* gene (Additional file 12: Table S3a) was injected into *cebp-1(tm2807)* worms, in mixtures composed of 50 ng/μl of *nipi-3* sgRNA, 30 ng/μl of *eft-3p::Cas9-SV40NLS::tbb-2 3′UTR* and 30 ng/μl of *col-12p::dsRed* [49]*.* Note that we also injected many wild-type worms with various combinations of *nipi-3* sgRNAs*,* but failed to isolate any deletion alleles.

GFP KI in the *nipi-3* locus, *nipi-3(fr152)* was generated with the same mixture as for *nipi-3(fr148)*, providing 30 ng/μl of the *nipi-3* repair template pSO1. This template was generated by Gibson assembly in the self-excising (SEC) cassette containing vector pDD282 [50] with 716 bp and 518 bp homology arms upstream and downstream of the *nipi-3* start codon, respectively (Additional file 12: Table S3a).

### Genetic screen for *nipi-3(0)* lethality suppressors

*nipi-3(ju1293)* mutant animals carrying *juEx6807[nipi-3 genomic DNA; myo-2p::mCherry]* were mutagenized using 45 mM ethyl methane sulphonate (EMS) following standard procedures as described [46]. Animals were distributed onto nematode growth media (NGM) plates seeded with *Escherichia coli* OP50 and screened in the F_2_ generation for normal animal growth reaching adulthood without expressing the transgene (no pharyngeal mCherry) under a fluorescence dissecting microscope.

### Mapping and cloning of *nipi-3* suppressor alleles

We first performed a conventional Sanger sequencing analysis for all suppressor alleles for *cebp-1* and determined that the *ju1367* allele affected *cebp-1.* We next sequenced *mak-2*, which was previously known to act in the same pathway as *cebp-1* in neurons [22], and found *ju1349* and *ju1352* alleles to affect *mak-2.* All other suppressors were analysed by whole genome sequencing analysis and single-nucleotide polymorphism (SNP) mapping following established methods [51]. Briefly, genomic DNA was prepared using a Puregene Cell and Tissue Kit (Qiagen) according to the manufacturer’s instructions, and 20X coverage of sequences was obtained using a 90 bp paired-end Illumina HiSeq 2000 at Beijing Genomics Institute (BGI Americas). The raw sequences were mapped to the *C. elegans* reference genome (WS220/ce10) using Burrows-Wheeler Aligner **(**BWA) [52] in the Galaxy platform (http://usegalaxy.org) [53]. Following subtraction of the nucleotide variants in the original strains, we generated a list of candidate genes containing unique homozygous nucleotide variants that were predicated to alter the function of the gene. We then confirmed the causality of the candidate genes by testing the known null alleles on the suppression of *nipi-3(0)*.

### Body length analysis

To examine the body length of animals during development, we obtained synchronized animals. Briefly, 5–15 gravid adults were placed on a seeded NGM plate to allow egg-laying for 3 h. Eggs laid during this period were incubated at 20 °C for 72 h, and the animals were then mounted on 2 % agarose pads containing a drop of 2.5 mM levamisole and photographed with a Zeiss Axioplan compound microscope, using Nomarski-DIC optics and an attached AxioCam digital camera. ImageJ software (National Institutes of Health (NIH), Bethesda, MD, USA) was used to measure body length by drawing a freehand midline from the tip of the nose to the tip of the tail of each animal.

### Western blot analysis

Worms of each genotype (80–100 individuals of L2 stage worms) were collected and washed with M9 buffer and boiled in sodium dodecyl sulphate (SDS) sample buffer for 10 min and loaded onto SDS-polyacrylamide gel electrophoresis (PAGE) gel (Bio-Rad). A 1:500 dilution of rabbit anti-phospho-p38 MAPK (Cell Signaling, #9211) and a 1:10,000 dilution of mouse anti-actin (MPbio, #08691001) were used as primary antibodies. ImageJ was used to quantify the intensity of the immunoblot bands.

### Quantitative RT-PCR

Quantitative real-time PCR was performed as previously described [49]. Sequences of primers are given in Additional file 12: Table S3b. To collect synchronized *nipi-3(0)* homozygous animals, we maintained *nipi-3(0)* animals on *cebp-1* RNAi plates. Gravid adults were then treated by bleaching solution to collect *nipi-3(0)* embryos, which were placed directly on regular NGM plates for 2 days in parallel to other strains.

### Fluorescence microscopy and axon regeneration by laser axotomy

Animals were mounted on 2 % agarose pads and immobilized with 2.5 mM levamisole. For transcriptional *cebp-1p::GFP*, GFP expression was imaged with a Zeiss Axioplan compound microscope, using Nomarski optics and an attached AxioCam digital camera. Translational CEBP-1::GFP expression was imaged with a Zeiss LSM710 confocal microscope for quantitative analyses. For confocal images, z stacks were obtained and maximum projection images were created using Zeiss Zen 2012 software. ImageJ was used to measure the GFP intensity at the nerve ring area for *cebp-1p::CEBP-1::GFP* and at each nucleus (10 per animal) for *col-154p::CEBP-1::GFP*. GFP::NIPI-3 KI expression was imaged with a spinning disk confocal microscope as described [54] to improve the signal-to-noise ratio.

We cut PLM axons and quantified the length of regrown axons as described [55].

### CEBP-1 ChIP-seq analysis

We generated transgenic animals expressing a functional FLAG-tagged CEBP-1 protein in a *cebp-1(tm2807)* mutant background (*cebp-1(0); juIs418 [cebp-1p::FLAG::CEBP-1::cebp-1 3′UTR]*) (Additional file 13: Figure S10) and then immunoprecipitated FLAG-CEBP-1-associated DNA fragments using anti-FLAG antibodies (M2 anti-FLAG magnetic beads; Sigma). We collected mixed stage worms grown at 20 °C on NGM plates followed by 2 % formaldehyde and sonicated the samples as described [56]. We next generated ChIP-seq DNA libraries via ligating DNA to specific adaptors and amplification with barcode primers, then sequenced them on the Illumina HiSeq 2000 platform. We performed two independent ChIP-seq experiments with parallel genomic DNA controls prepared from the same strain. We conducted peak calling using a CLC Genomics Workbench 6.0 (CLC bio). To define genes associated with the peaks, we used the annotation of transcription start site (TSS) and transcription end site (TES) from Wormbase WS220 (http://www.wormbase.org) and annotated the peak if it overlapped with the gene or the 3 kb upstream of the TSS. We then manually confirmed the peaks and associated genes using the University of California, Santa Cruz (UCSC) browser and an update to WS252. If the peak was found within the promoter for one isoform and introns for other isoforms, we categorized it as a peak within a promoter. The ChIP-seq data are available at the Gene Expression Omnibus [GEO:GSE83330].

### Bioinformatic analyses

MEME [33] and RSAT [34] were used to identify over-represented motifs from 209 CEBP-1 ChIP-seq peak sequences, and then Tomtom [35] was used to compare the most over-represented motif against a database of known motifs in vertebrates. All informatics tools can be found at http://meme-suite.org and http://www.rsat.eu. Enrichment analyses were run on a newly developed database of functional annotations including 4600 datasets [39] updated to Wormbase WS252 (http://www.wormbase.org). Classes considered for enrichment had a maximum size of 2000 genes, and the *P* value for enrichment was lower than 10^-5^.

### Yeast two-hybrid assay

Full-length or fragments of complementary DNAs (cDNAs) were cloned into the pACT2 (Gal4 activation domain) or pBTM116 (LexA DNA-binding domain) vectors (Clontech) and constructs were co-transformed into yeast strain L40. We grew transformed yeasts on agar plates with synthetic defined (SD) minimal medium lacking leucine and tryptophan; interactions were examined on plates with SD medium lacking leucine, tryptophan and histidine.

### Statistical analysis

Statistical analysis was performed using GraphPad Prism 5. Significance was determined using unpaired *t* tests for two samples, one-way ANOVA followed by Tukey’s multiple comparison tests for multiple samples. For two nominal variables, Fisher’s exact test was used to evaluate the statistical significance. *P* < 0.05 (*) was considered statistically significant (* P < 0.05; ** P < 0.01; *** P < 0.001).

## Additional files

Additional file 1: Figure S1. CEBP-1 expression in multiple tissues causes an abnormal pharyngeal morphology in *nipi-3(0) cebp-1(0)* animals. (a) Differential interference contrast (*DIC*) images of worms at 3 days post-hatching. Pharynx and intestine are denoted by a *dotted red* and *yellow line*, respectively. (b) Co-expression of *cebp-1(+)* in the epidermis and neurons in *nipi-3(0) cebp-1(0)* animals caused pharyngeal morphology defect. **P* < 0.05; ***P* < 0.01; *ns*, not significant (two-tailed Fisher’s exact test). (PDF 3776 kb)
Additional file 2: Figure S2. NIPI-3 interacts with CEBP-1 in yeast two-hybrid assay. CEBP-1 variants were fused to the Gal4 activation domain and tested for their interaction with full-length NIPI-3 fused to the LexA DNA-binding domain. A large CEBP-1 fragment (∆2, amino acids 1–115) was sufficient for the NIPI-3 interaction. – refers to no growth and + refers to growth in the absence of histidine. (PDF 433 kb)
Additional file 3: Figure S3. Loss of *cebp-1* rescues the *nipi-3(fr4)* phenotype. (a) Bright-field images and (b) body length of worms at 3 days post-hatching grown at 25 °C. (b) Each *dot* represents a single animal measured as shown; each *red line* represents the mean value. ****P* < 0.001; *ns*, not significant (one-way ANOVA with Tukey’s post hoc tests). (PDF 1253 kb)
Additional file 4: Figure S4.
*cebp-1* and *nipi-3* are dispensable in immune response and axon regeneration, respectively. (a) qRT-PCR analysis of AMP gene, *nlp-34.* Relative abundance of *nlp-34* mRNA normalized to *actin* mRNA. *n =* 3. Error bars represent SEM. ****P* < 0.001; *ns*, not significant (one-way ANOVA with Tukey’s post hoc tests). Primary data are provided in Additional file 14. (b) Axotomy and axon regeneration analysis. Normalized PLM axon regrowth is shown in the bar graph. Error bars represent SEM. ****P* < 0.001; *ns*, not significant (one-way ANOVA with Tukey’s post hoc tests). (PDF 472 kb)
Additional file 5: Figure S5. Quantification of the body length of suppressor alleles identified from the screen and loss-of-function mutants of *tpa-1, pmk-1, sta-2, dlk-1, pmk-3, mlk-1* and *kgb-1*. (a, b) Body length of worms at 3 days post-hatching. Each *dot* represents a single animal measured as shown; each *red line* represents the mean value; some data are replicated from Fig. 1 as shown with *darker grey dots*. ****P* < 0.001 (one-way ANOVA with Tukey’s post hoc tests). (PDF 569 kb)
Additional file 6: Figure S6. Phosphorylated PMK-1 levels are unchanged in *nipi-3(0); mak-2(0)* animals. (a) Western blot analysis on total protein lysate from various animal strains using the indicated antibodies, α-phospho-p38 MAPK antibody to detect a phosphorylated form of PMK-1 proteins (*p-PMK-1*) or α-actin antibody as a loading control. (b) Densitometric quantifications of immunoblot signals normalized to actin. *n =* 4; error bars represent SEM*; ns*, not significant (Student’s paired *t* test). Primary data are provided in Additional file 14. (PDF 595 kb)
Additional file 7: Figure S7.
*cebp-1* shows a dosage sensitive effect in *nipi-3(0)* mutants*.* (a) Bright-field images and (b) body length of worms at 3 days post-hatching. (b) Each *dot* represents a single animal measured as shown; each *red line* represents the mean value. ****P* < 0.001 (one-way ANOVA with Tukey’s post hoc tests). (PDF 1501 kb)
Additional file 8: Table S1. List of CEBP-1 target genes and hierarchical clustering of genes and functional classes (XLSX 64 kb)
Additional file 9: Figure S8. The promoter of *sek-1* contains a ChIP-seq peak of CEBP-1*.* The promoter region of *sek-1* contains two consensus DNA-binding motifs for CEBP-1 (*black triangles*). *Top*, the *sek-1* locus. *Middle*, sequencing reads from CEBP-1-IP. *Bottom*, sequencing reads from genomic DNA input. (PDF 253 kb)
Additional file 10: Figure S9. Hierarchical clustering of genes and functional classes. The presence of a gene in a class is represented by a *red rectangle*, its absence in *blue*. See Additional file 8: Table S1 for class labels and full data. (PDF 680 kb)
Additional file 11: Table S2. List of strains and alleles. (XLSX 18 kb)
Additional file 12: Table S3. List of plasmids and primers used for qRT-PCR. (XLSX 13 kb)
Additional file 13: Figure S10. FLAG-tagged CEBP-1 protein rescues PLM axon regeneration defects of *cebp-1(0).* Axotomy and axon regeneration analysis. Normalized PLM axon regrowth is shown in the bar graph. Error bars represent SEM. ****P* < 0.001; *ns*, not significant (one-way ANOVA with Tukey’s post hoc tests). (PDF 432 kb)
Additional file 14: Primary data for Figs. 3b, c, k and 5b and Figures S4a and S6b. (XLSX 20 kb)

## Abbreviations

AMP: Antimicrobial peptide
bZIP: Basic leucine zipper
C/EBP: CCAAT/enhancer-binding protein
ChIP-seq: Chromatin immunoprecipitation and deep sequencing
CRISPR: Clustered regularly interspaced short palindromic repeats
EASE: Expression Analysis Systematic Explorer
GO: Gene ontology
KEGG: Kyoto Encyclopedia of Genes and Genomes
MAPK: Mitogen-activated protein kinase
MEME: Multiple Em for Motif Elicitation
NIPI: No induction of peptide after *Drechmeria* infection
RSAT: Regulatory Sequence Analysis Tools
