# Supplementary material for: Coordinated inhibition of C/EBP by Tribbles in multiple tissues is essential for Caenorhabditis elegans development
Source: BMC Biol. 2016 Dec 7;14:104. doi: 10.1186/s12915-016-0320-z (PMC5141650; doi:10.1186/s12915-016-0320-z)
Supplement: Additional file 1: Figure S1. — CEBP-1 expression in multiple tissues causes an abnormal pharyngeal morphology in nipi-3(0) cebp-1(0) animals. (a) Differential interference contrast (DIC) images of worms at 3 days post-hatching. Pharynx and intestine are denoted by a dotted red and yellow line, respectively. (b) Co-expression of cebp-1(+) in the epidermis and neurons in nipi-3(0) cebp-1(0) animals caused pharyngeal morphology defect. *P < 0.05; **P < 0.01; ns, not significant (two-tailed Fisher’s exact test). (PDF 3776 kb) [file 12915_2016_320_MOESM1_ESM.pdf]

Figure S1

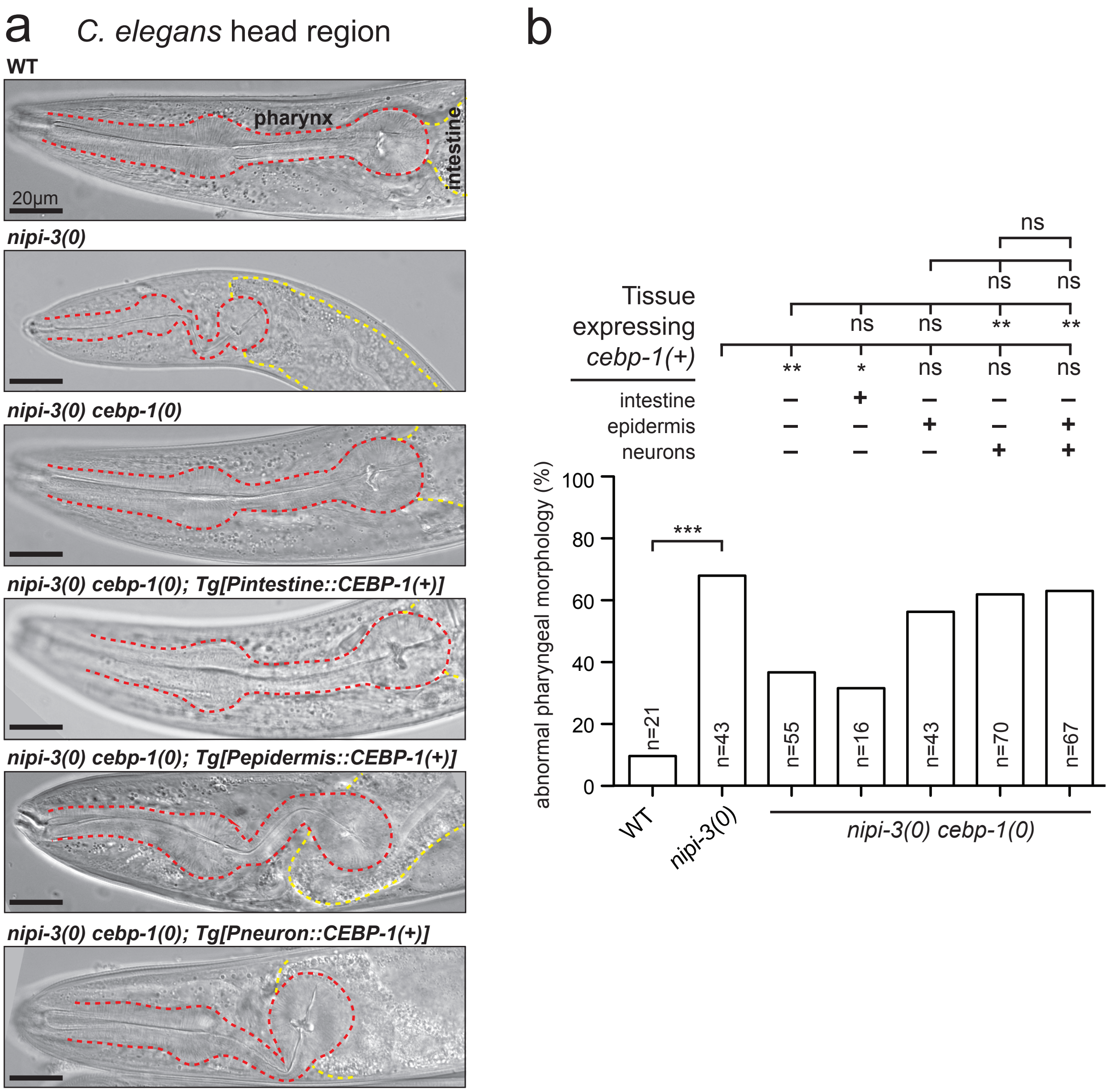

a
C. elegans head region
WT
pharynx
intestine
20µm
nipi-3(0)
nipi-3(0) cebp-1(0)
nipi-3(0) cebp-1(0); Tg[Pintestine::CEBP-1(+)]
nipi-3(0) cebp-1(0); Tg[Pepidermis::CEBP-1(+)]
nipi-3(0) cebp-1(0); Tg[Pneuron::CEBP-1(+)]
b
Tissue expressing cebp-1(+)
intestine
epidermis
neurons
ns
ns ns
ns ns ** **
** * ns ns ns
***
abnormal pharyngeal morphology (%)
100
80
60
40
20
0
n=21
n=43
n=55
n=16
n=43
n=70
n=67
WT
nipi-3(0)
nipi-3(0) cebp-1(0)
