# Supplementary figures and images for: Coordinated inhibition of C/EBP by Tribbles in multiple tissues is essential for Caenorhabditis elegans development
Source: BMC Biol. 2016 Dec 7;14:104. doi: 10.1186/s12915-016-0320-z (PMC5141650; doi:10.1186/s12915-016-0320-z)

Figure S2

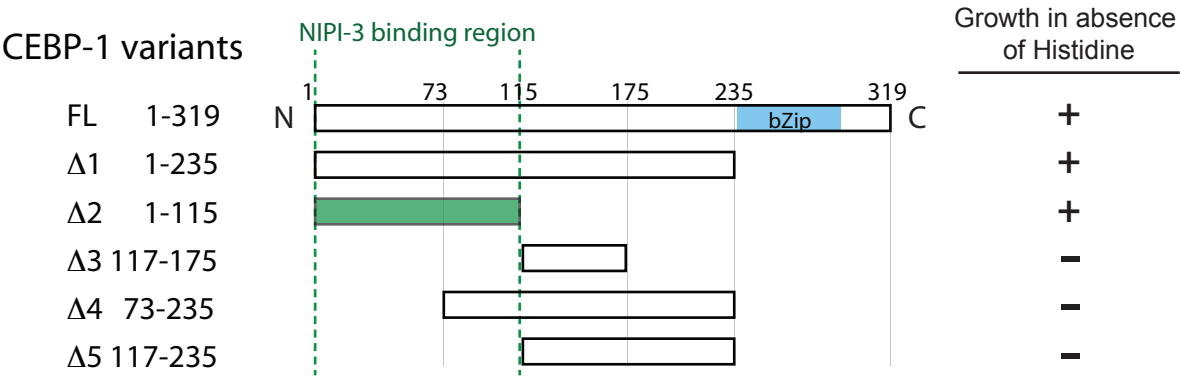

Supplement: Additional file 2: Figure S2. — NIPI-3 interacts with CEBP-1 in yeast two-hybrid assay. CEBP-1 variants were fused to the Gal4 activation domain and tested for their interaction with full-length NIPI-3 fused to the LexA DNA-binding domain. A large CEBP-1 fragment (∆2, amino acids 1–115) was sufficient for the NIPI-3 interaction. – refers to no growth and + refers to growth in the absence of histidine. (PDF 433 kb) [file 12915_2016_320_MOESM2_ESM.pdf]

Figure S3

a

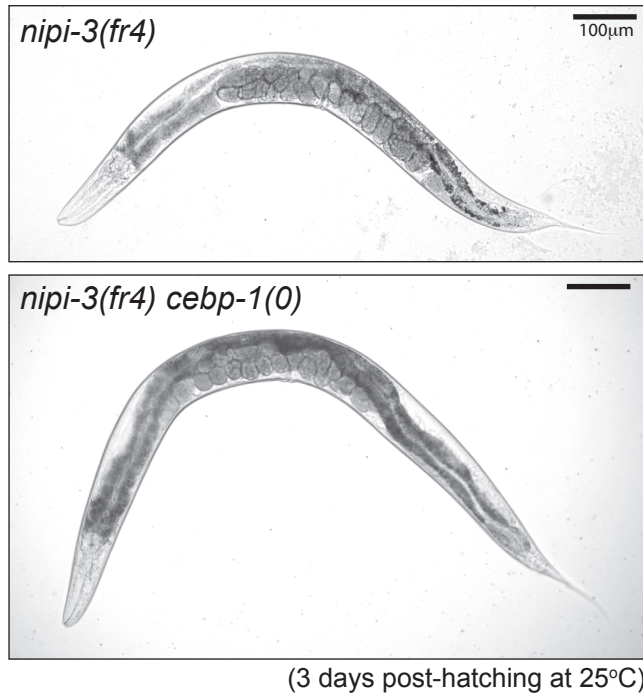

b

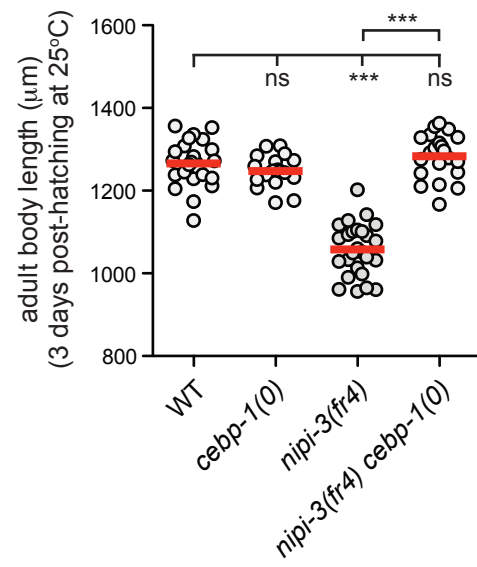

Supplement: Additional file 3: Figure S3. — Loss of cebp-1 rescues the nipi-3(fr4) phenotype. (a) Bright-field images and (b) body length of worms at 3 days post-hatching grown at 25 °C. (b) Each dot represents a single animal measured as shown; each red line represents the mean value. ***P < 0.001; ns, not significant (one-way ANOVA with Tukey’s post hoc tests). (PDF 1253 kb) [file 12915_2016_320_MOESM3_ESM.pdf]

**Figure S4**

**a** *nlp-34* induction after fungal infection

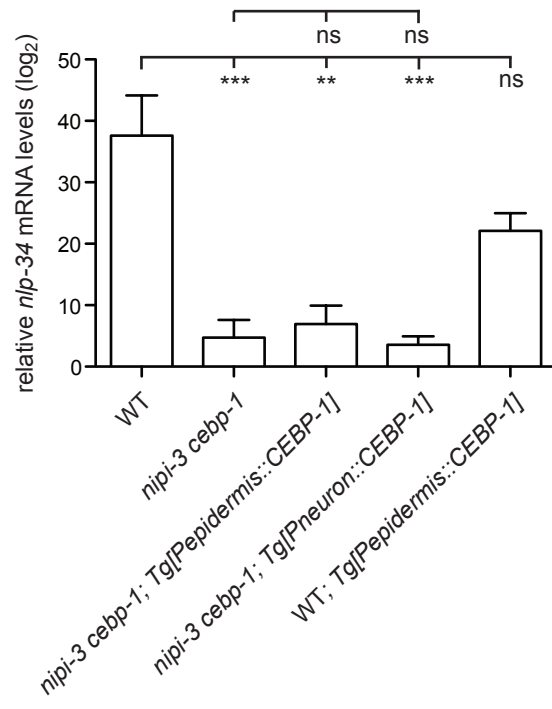

**b**

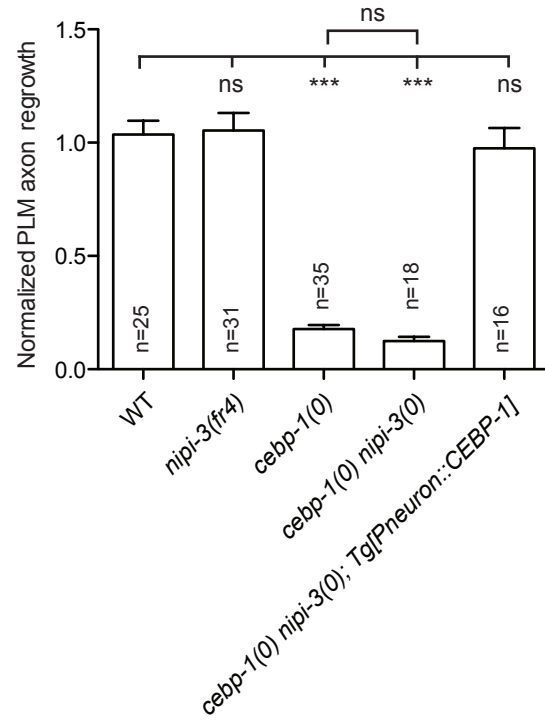

Supplement: Additional file 4: Figure S4. — cebp-1 and nipi-3 are dispensable in immune response and axon regeneration, respectively. (a) qRT-PCR analysis of AMP gene, nlp-34. Relative abundance of nlp-34 mRNA normalized to actin mRNA. n = 3. Error bars represent SEM. ***P < 0.001; ns, not significant (one-way ANOVA with Tukey’s post hoc tests). Primary data are provided in Additional file 14. (b) Axotomy and axon regeneration analysis. Normalized PLM axon regrowth is shown in the bar graph. Error bars represent SEM. ***P < 0.001; ns, not significant (one-way ANOVA with Tukey’s post hoc tests). (PDF 472 kb) [file 12915_2016_320_MOESM4_ESM.pdf]

Figure S5

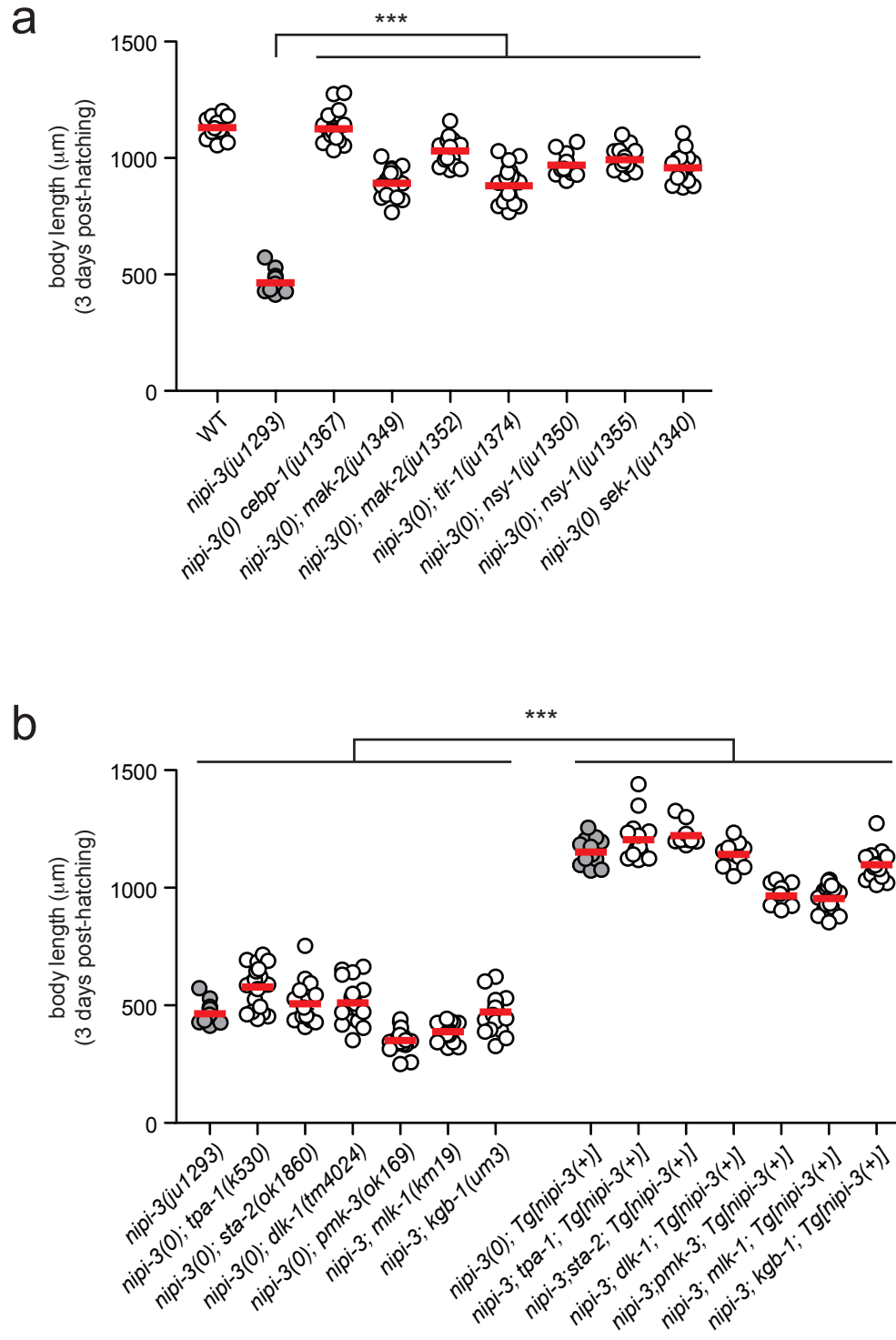

Supplement: Additional file 5: Figure S5. — Quantification of the body length of suppressor alleles identified from the screen and loss-of-function mutants of tpa-1, pmk-1, sta-2, dlk-1, pmk-3, mlk-1 and kgb-1. (a, b) Body length of worms at 3 days post-hatching. Each dot represents a single animal measured as shown; each red line represents the mean value; some data are replicated from Fig. 1 as shown with darker grey dots. ***P < 0.001 (one-way ANOVA with Tukey’s post hoc tests). (PDF 569 kb) [file 12915_2016_320_MOESM5_ESM.pdf]

**Figure S6**

**a**

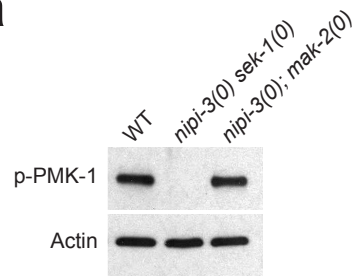

**b**

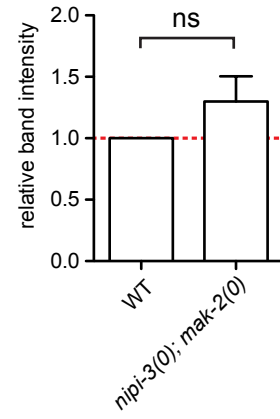

Supplement: Additional file 6: Figure S6. — Phosphorylated PMK-1 levels are unchanged in nipi-3(0); mak-2(0) animals. (a) Western blot analysis on total protein lysate from various animal strains using the indicated antibodies, α-phospho-p38 MAPK antibody to detect a phosphorylated form of PMK-1 proteins (p-PMK-1) or α-actin antibody as a loading control. (b) Densitometric quantifications of immunoblot signals normalized to actin. n = 4; error bars represent SEM; ns, not significant (Student’s paired t test). Primary data are provided in Additional file 14. (PDF 595 kb) [file 12915_2016_320_MOESM6_ESM.pdf]

**Figure S7**

**a**

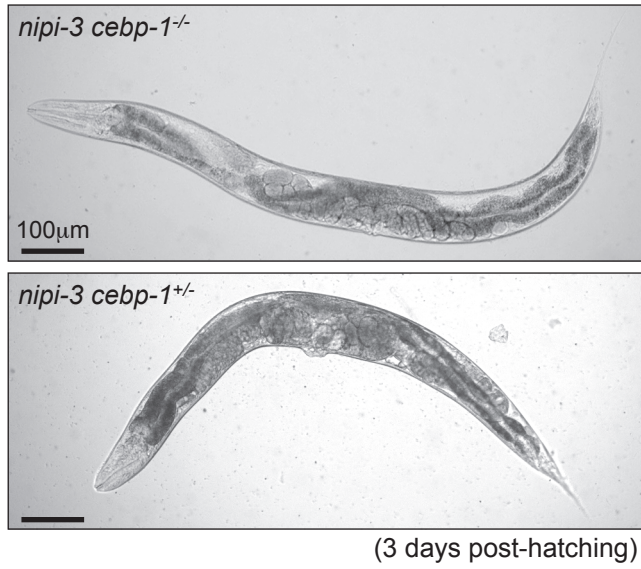

**b**

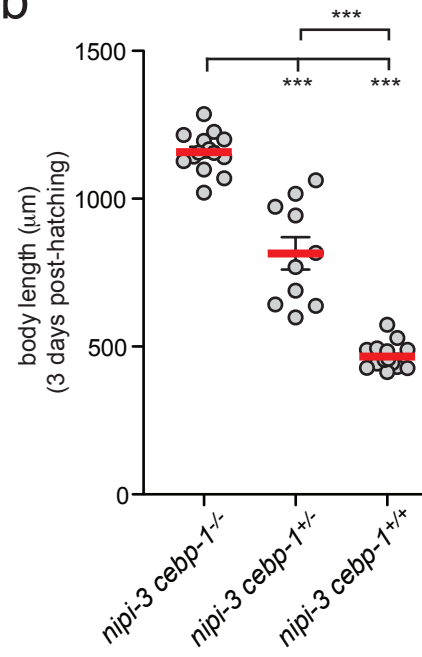

Supplement: Additional file 7: Figure S7. — cebp-1 shows a dosage sensitive effect in nipi-3(0) mutants. (a) Bright-field images and (b) body length of worms at 3 days post-hatching. (b) Each dot represents a single animal measured as shown; each red line represents the mean value. ***P < 0.001 (one-way ANOVA with Tukey’s post hoc tests). (PDF 1501 kb) [file 12915_2016_320_MOESM7_ESM.pdf]

Figure S8

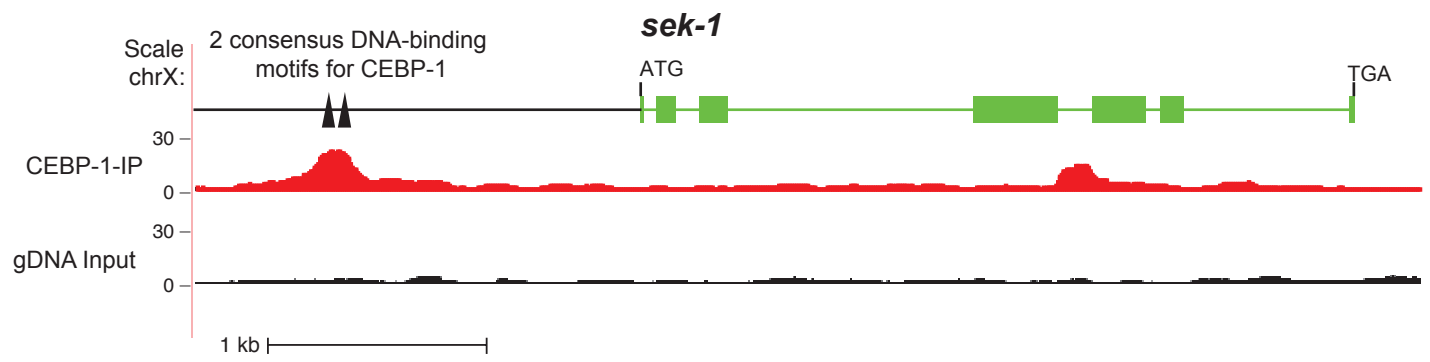

Supplement: Additional file 9: Figure S8. — The promoter of sek-1 contains a ChIP-seq peak of CEBP-1. The promoter region of sek-1 contains two consensus DNA-binding motifs for CEBP-1 (black triangles). Top, the sek-1 locus. Middle, sequencing reads from CEBP-1-IP. Bottom, sequencing reads from genomic DNA input. (PDF 253 kb) [file 12915_2016_320_MOESM9_ESM.pdf]

Figure S9

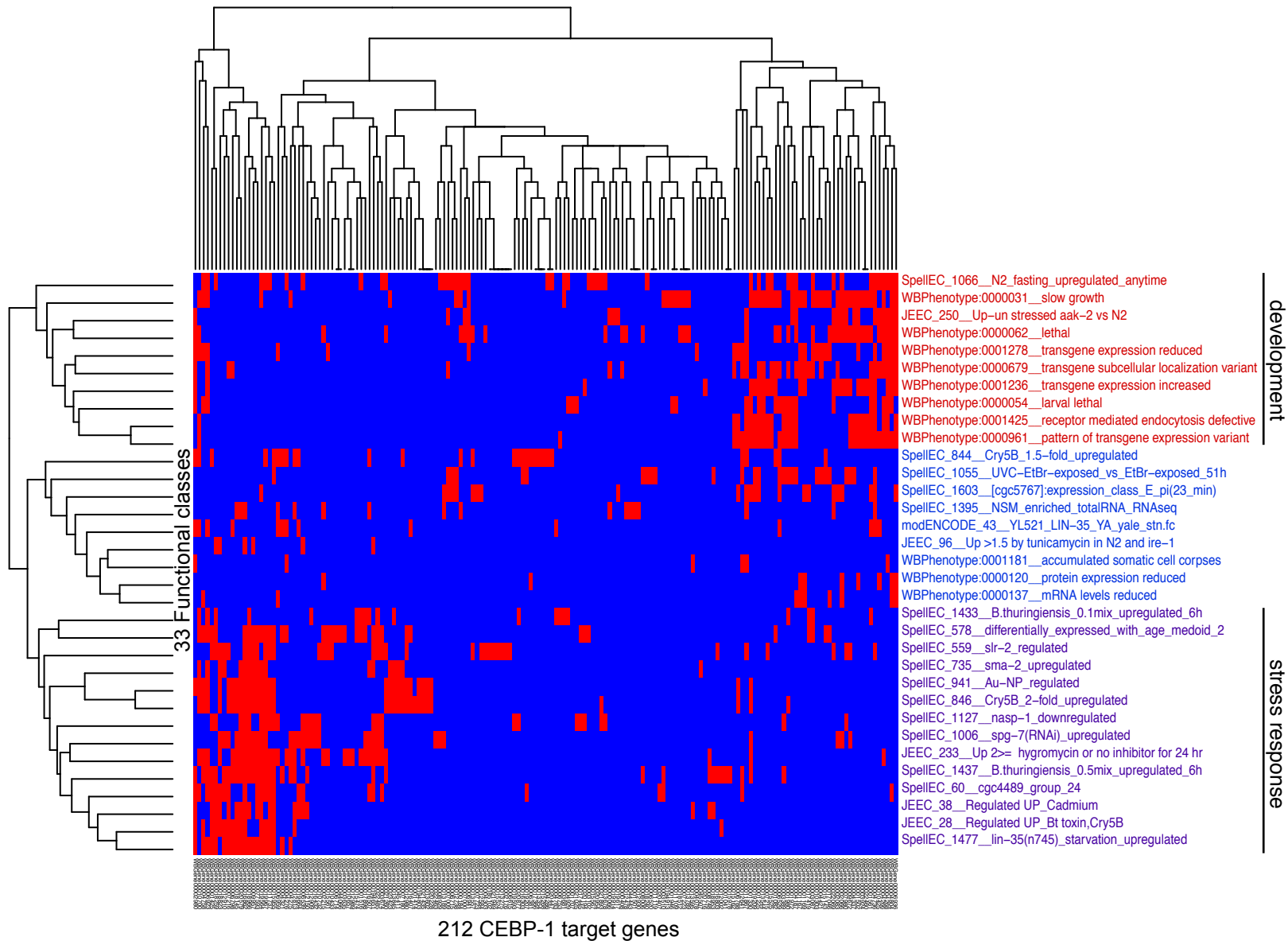

Supplement: Additional file 10: Figure S9. — Hierarchical clustering of genes and functional classes. The presence of a gene in a class is represented by a red rectangle, its absence in blue. See Additional file 8: Table S1 for class labels and full data. (PDF 680 kb) [file 12915_2016_320_MOESM10_ESM.pdf]

**Figure S10**

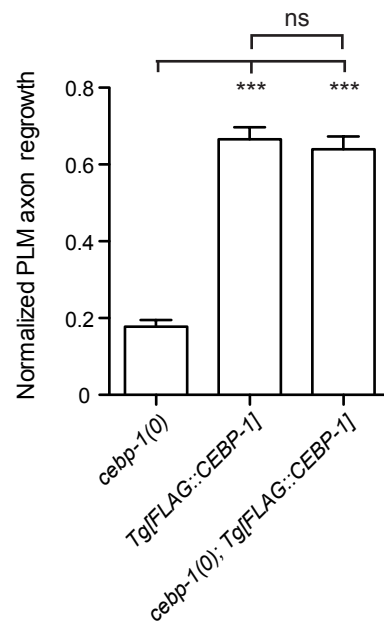

Supplement: Additional file 13: Figure S10. — FLAG-tagged CEBP-1 protein rescues PLM axon regeneration defects of cebp-1(0). Axotomy and axon regeneration analysis. Normalized PLM axon regrowth is shown in the bar graph. Error bars represent SEM. ***P < 0.001; ns, not significant (one-way ANOVA with Tukey’s post hoc tests). (PDF 432 kb) [file 12915_2016_320_MOESM13_ESM.pdf]
